# An Index of Surgery: Being a Concise Classification of the Main Facts and Theories of Surgery, for the Use of Senior Students and Others

**Published:** 1887-12

**Authors:** 


*An Index of Surgery: being a Concise Classification of the Main Facts and Theories of Surgery, for the use of Senior Students and others.* By C. B. KEETLEY, F.R.C.S. Fourth Edition. London: SMITH, ELDER, & Co. 1887.

Praise would be impertinent in the case of the fourth edition of so well-known a book as Keetley's *Index.* Although primarily intended for students, it seems more valuable as a book of notes for everyday reference. For fuller information, it supplies copious references to surgical literature.

The book invites perusal by its clear and well-printed characters. The proof-reading seems to have been done carefully; but at p. 311 "glycerina" is wrong, and at p. 519 and in the index "Geath" should be "Genth." At p. 220 there is, in the text, a reference to a non-existent foot-note.

